# A polar Fourier geometric approach to volume-free single-particle 3D reconstruction

**DOI:** 10.1107/S2052252526003611

**Published:** 2026-06-11

**Authors:** Cong T. S. Van, Cyril F. Reboul, Joseph J. E. Caesar, Rubén Meana-Pañeda, Hans Elmlund

**Affiliations:** a National Cancer Institute (NCI) National Institutes of Health (NIH) Frederick MD21701 USA; Max Planck Institute of Molecular Physiology, Germany

**Keywords:** single-particle 3D reconstruction, polar Fourier coordinates, orientation-recovery problem

## Abstract

We introduce a compact mathematical formulation for the inverse single-particle 3D reconstruction problem, a high-dimensional inverse problem in which millions of parameters are estimated from extremely noisy experimental measurements. Given a collection of noisy 2D projection images (particles) of an unknown 3D charge-density distribution, the objective is to infer the unknown particle orientations and thereby enable *ab initio* 3D reconstruction via tomographic methods. We develop a method for generating regularized reprojections directly from the noisy particles that does not rely on explicit 3D density reconstruction. Instead, we recast the *ab initio* orientation-recovery problem in polar Fourier coordinates through discretization of the rotation group SO(3). The directions of projection are mapped onto slices intersecting the origin of the 3D Fourier transform. The rotations in the plane normal to a projection direction are mapped onto radial lines in the 2D Fourier transforms of the particles. An optimization procedure jointly estimates particle projection directions, in-plane rotation angles and rotational origin offsets. Regularized reprojections are computed by averaging along lines in the polar Fourier representation, exploiting data redundancy to suppress noise and improve stability. We present the mathematical framework in detail and provide initial benchmarks demonstrating the performance and robustness of the approach.

## Abstract 2

We present a compact mathematical framework for *ab initio* single-particle 3D reconstruction that reformulates the inverse problem in polar Fourier coordinates, enabling direct orientation recovery from extremely noisy 2D projection images without explicit 3D density reconstruction.

## 1. Introduction

The fundamental theory for 3D reconstruction from projection images in electron microscopy was developed by De Rosier & Klug (1968 ▸) and used to reconstruct the tomato bushy stunt virus (Crowther *et al.*, 1970 ▸). The theory is based on the projection-slice theorem (Bracewell, 1956 ▸), which states that the Fourier transform of the projection of a 3D charge-density distribution corresponds to a central section through the 3D volume’s Fourier transform. The projection-slice theorem underpins convolution interpolation in electron microscopy, both for extracting reprojections from volumes at known 3D orientations (Yang & Penczek, 2008 ▸; Penczek, 2010 ▸; Penczek *et al.*, 2004 ▸) and for calculating 3D reconstructions from particles with assigned 3D orientations (Penczek *et al.*, 2004 ▸; O’Sullivan, 1985 ▸; Beatty *et al.*, 2005 ▸; Jackson *et al.*, 1991 ▸). Another consequence of the projection-slice theorem is that any two projection images with non-parallel directions will share a common line in the Fourier domain, and their relative 3D orientations will be fixed to a rotation around the axis defined by this line. This concept of ‘common lines’ provided the mathematical foundation for angular reconstitution (Van Heel, 1987 ▸), where the relative 3D orientations of three class averages are determined unambiguously and used as an anchor set to determine the relative 3D orientations of larger sets of class averages. Angular reconstitution was the first *ab initio* 3D reconstruction approach used to successfully reconstruct particles with low point-group symmetry (Stark *et al.*, 1995 ▸; Schatz *et al.*, 1995 ▸). However, no *ab initio* 3D reconstruction approach can directly resolve the ambiguity of enantiomorphism. Therefore, the handedness of the 3D density map must be determined from the handedness of resolved secondary structure elements, as it was done for the bacteriorhodopsin electron crystallography structure (Henderson *et al.*, 1990 ▸), or tilt experiments if the resolution of the 3D map is insufficient (Rosenthal & Henderson, 2003 ▸).

The theory of common lines has remained in use for icosahedral particles (Fuller *et al.*, 1996 ▸) but modern probabilistic *ab initio* 3D reconstruction approaches (Zivanov *et al.*, 2018 ▸; Punjani *et al.*, 2017 ▸; Van *et al.*, 2025 ▸) instead rely on projection matching (Grigorieff, 2007 ▸; Penczek *et al.*, 1992 ▸), where particles are iteratively matched with volume reprojections to generate probabilities that direct the orientation search. Much of the recent work on 3D reconstruction theory utilizing common lines has focused on improving their error-prone detection (Hall *et al.*, 2007 ▸; Singer *et al.*, 2010 ▸; Singer & Shkolnisky, 2011 ▸; Herman & Kalinowski, 2008 ▸; Wang *et al.*, 2024 ▸) or the use of algebraic constraints for denoising prior to detection (Muller *et al.*, 2024 ▸).

Single-particle 3D reconstruction algorithms based on probabilistic inference typically define the model being refined as the real-space volumetric density sought (Zhong *et al.*, 2021 ▸; Punjani *et al.*, 2017 ▸; Jaitly *et al.*, 2010 ▸; Scheres, 2012*a* ▸; Lyumkis *et al.*, 2013 ▸). However, the model required for generating reprojections with maximally enhanced signal-to-noise ratio (SNR) does not have to match the model that we seek to estimate from a structural point of view. In this study, we therefore asked whether the concept of common lines could be used to create an abstract mathematical model that replaces volumetric 3D reconstruction, while preserving the desired signal enhancement along equivalent lines in the dataset. All computations related to generating and manipulating the 3D reconstruction (masking, filtering, reprojection, *etc*.) are removed, and an algorithm with computational complexity similar to 2D multi-reference alignment (Scheres, Valle & Carazo, 2005 ▸; Scheres, Valle, Nuñez *et al.*, 2005 ▸; Reboul *et al.*, 2016 ▸; Yang *et al.*, 2012 ▸; Sorzano *et al.*, 2010 ▸; Romanov *et al.*, 2021 ▸) is provided that expresses the geometrical constraints inherent to the system – that non-parallel pairs of reprojections share a common line in the Fourier domain. Our mathematical model embeds a search space of *N*_P_ projection directions, which are equidistant points on the unit sphere. The *N* particles are assigned to these *N*_P_ projection directions, while optimizing the in-plane degrees of freedom. When creating an average from each projection direction, the common lines from particles in the other *N*_P_ − 1 projection directions each contribute a single intersecting line to the generation of the ‘class average’, which is more appropriately referred to as a ‘reprojection estimate’ in this setting. Unlike classical common-line approaches, which attempt to explicitly detect and validate individual common lines in noisy data, our method assumes a discretized orientation manifold and uses common-line geometry implicitly as a deterministic averaging operator. Hence, common lines are not detected but enforced geometrically through SO(3) discretization. We simply assume that the projection directions in the dataset data are distributed according to some distribution on the unit sphere that defines the geometrical relationships needed for averaging. Our formulation replaces explicit 3D Fourier gridding with an operator defined directly on discretized central sections of reciprocal space, preserving the projection-slice geometry while eliminating the need for volumetric interpolation. Optimization of the model can be carried out exactly as it is done for projection-matching-based implementations (Grigorieff, 2007 ▸; Scheres, 2012*a* ▸; Punjani *et al.*, 2017 ▸; Van *et al.*, 2025 ▸) or multi-reference 2D alignment (Reboul *et al.*, 2016 ▸; Scheres, Valle, Nuñez *et al.*, 2005 ▸; Sorzano *et al.*, 2010 ▸; Yang *et al.*, 2012 ▸; Sigworth, 1998 ▸; Romanov *et al.*, 2021 ▸). We implemented the method in polar coordinates; the particles are converted into 2D polar Fourier transforms and then reprojection estimates are generated through averaging lines using pre-calculated data structures that express the geometrical relationships. We outline the mathematical theory in detail, describe tests that validate the accuracy of our implementation, and provide benchmarks that demonstrate how the method can be used to accelerate projection-matching-based approaches for iterative *ab initio* 3D reconstruction.

## 2. Methods

### 2.1. Statistical formulation of the orientation-recovery problem

*Ab initio* 3D reconstruction in single-particle cryo-EM requires estimating unknown particle 3D orientations from extremely noisy 2D projection images. We consider a set of *N* noisy particles 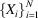, modeled in Fourier space as central sections of the Fourier transform of an unknown 3D density *V*. For particle *i*, the forward model is
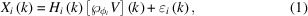
where *k* indexes 2D Fourier coordinates, 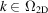; 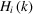 is the contrast transfer function (CTF); 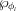 is the projection operator at pose 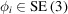 (3D rotation + in-plane shift); 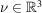 is the real-valued 3D density sought and *V* its Fourier transform; and 
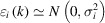
 is additive Gaussian noise.

We seek to estimate orientations 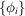 and volume *V* using the following regularized objective:
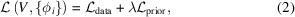
where 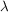 is a regularization parameter and
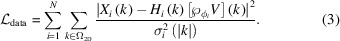
The regularization objective
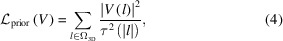
where *l* indexes 3D Fourier coordinates and 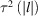 is a shell-wise signal variance, imposes a Gaussian prior on the reconstructed 3D Fourier coefficients
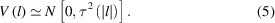


### 2.2. Volume-based fixed-point optimization

Standard fixed-point approaches alternate between estimating orientations by projection matching and reconstructing an intermediate 3D volume. While effective, this strategy is computationally expensive and enforces a tight coupling between orientation estimation and 3D volume interpolation. In contrast to the approach implemented in the software package *RELION* (Scheres, 2012*a* ▸,*b* ▸), which uses weighted orientation assignment, we use a hard orientation assignment optimization scheme, alternating between orientation update
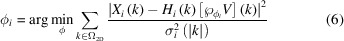
and volume update for fixed orientations:
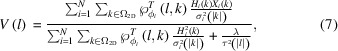
where 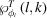 is the reconstruction operator that maps 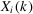 onto the 3D Fourier grid through convolution interpolation with a Kaiser–Bessel kernel (Penczek *et al.*, 2004 ▸; Beatty *et al.*, 2005 ▸). The summation over *k* in equation (7) represents summation over neighboring components of a central section of the Fourier volume. The result of the expression 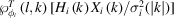 is a 3D window of Fourier components. Although a value of <1 was originally proposed for the regularization parameter 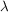 (Scheres, 2012*a* ▸,*b* ▸), we used 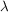 = 1 for all subsequent calculations. We previously described the probabilistic optimization algorithm used for solving equation (6) in the expectation-maximization iterations (Van *et al.*, 2025 ▸).

### 2.3. Proposed volume-free fixed-point reconstruction framework

**Figure 1:**
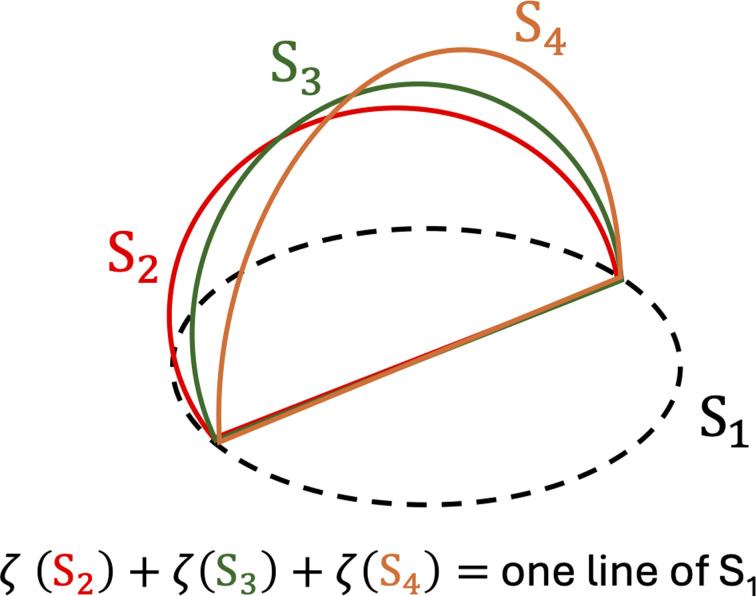
Illustration of reciprocal-space line contributions induced by the projection-slice theorem on discretized SO(3). One line of the common-line contribution on Fourier slice 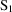 associated with orientation 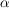 does not contain the line of 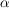 itself but contains the sum of all common lines between plane 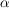 and slices 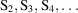 of 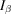.

**Figure 2:**
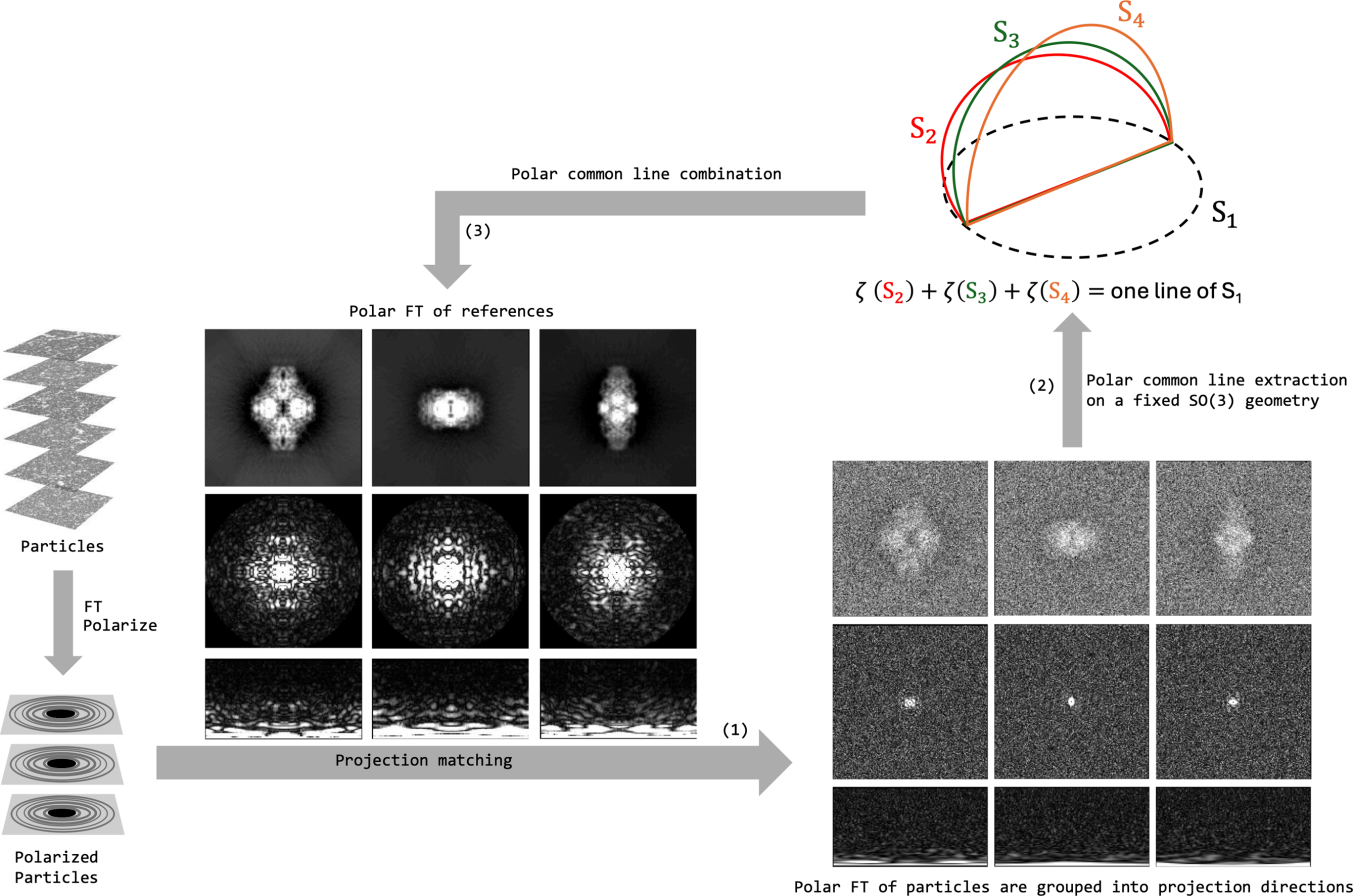
Schematic summary of our method for polar Fourier *ab initio* 3D reconstruction. Particles are Fourier transformed and converted to polar coordinates. At each iteration of expectation maximization, polar reprojection estimates are generated from the particles using the discrete SO(3) configuration obtained in the previous iteration. No 3D volume is needed or computed during the iterative process. A final 3D volume in Cartesian coordinates is reconstructed using the final relative 3D orientations assigned to the particles.

Here we introduce an alternative formulation in which orientation estimation is carried out entirely in the polar Fourier domain, without explicitly forming intermediate 3D volumes. The key idea is to replace volume-based reprojection with a linear operator that directly estimates reprojections from the set of noisy particles using discretized SO(3) geometry and polar common-line constraints. The SO(3) manifold is the mathematical space that represents all possible ways an object can be rotated in 3D space around a fixed center, whereas the 2-sphere (S^2^) is the surface of this 3D space at a defined radius. Furthermore, the S^1^ manifold denotes a circle representing all possible rotations about the origin in a single 2D plane. Our discretization of the rotation group SO(3) maps the 2-sphere, S^2^, onto slices passing through the origin of the 3D Fourier transform, and the circle manifold, S^1^, onto radial lines in the 2D Fourier transforms of the particles. We discretize 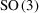 to a uniform grid of size 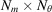 over 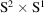, where 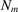 is the number of slices (references) on 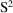 and 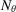 is the number of lines (in-plane rotations) on 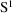, so each orientation 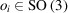 corresponds to 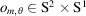. An Archimedean spiral on 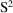 is used to generate evenly distributed projection directions (Baldwin & Penczek, 2007 ▸). Particles, CTF and σ^2^ factors are converted to polar Fourier coordinates:
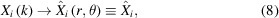

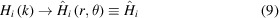
and
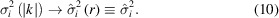
Equations (8)–(10) define the short-hand notation used from here on. In the polar representation, projection directions correspond to Fourier slices, in-plane rotations correspond to angular shifts, and common lines correspond to identical radial lines between slices. Instead of computing 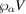 we construct reprojection estimates directly from particles assigned to orientations. We define the index set 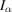 comprising all particles assigned to projection direction 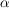. We also define the index set 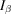 comprising all particles assigned to all orientations 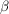 other than 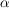: 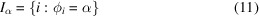
and
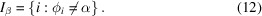
Next, we define a linear operator that estimates the reprojection at 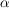 from the contributions of particles from both sets 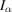 and 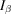. Formally, we define the operator as
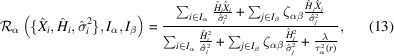
where 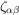 extracts and interpolates the common radial line. Hence, the result of the summation 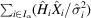 is the unnormalized 2D polar Fourier transform that would typically be referred to as a ‘class average’ in multi-reference 2D alignment, whereas the result of the summation 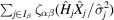 is the unnormalized 2D polar Fourier transform constructed from line contributions from particles assigned to different orientations. The 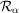 operator ties together the projection-slice theorem and the discretized angular search space through the discrete polar Fourier transform (Fig. 1 ▸). We replace the volume-based projection in the objective function to obtain the orientation-update equation in the volume-free framework. Thus,
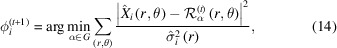
where *G* is the discretized SO(3) grid. This preserves the optimization objective but changes the representation of the reprojection operator. In contrast to volume-based projection matching, the common-lines formulation separates the contribution of lines from particles with parallel projection directions from the contribution of lines from particles with non-parallel directions in the reprojection estimation. This allows us to address the problem of directional bias (Reuter & Fischl, 2011 ▸) with the updated reprojection estimation operator
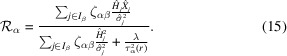
Equation (15) corresponds to replacing the full reprojection estimator in equation (13) with a leave-one-direction-out estimator, mirroring other common-lines-based approaches (Elmlund & Elmlund, 2009 ▸; Elmlund *et al.*, 2010 ▸, 2008 ▸). Since particles assigned to identical projection directions share no geometrically independent common lines, excluding them does not remove independent constraints. The final volume is reconstructed in Cartesian coordinates by equation (7) after all 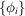 have been estimated using equations (14) and (15). Fig. 2 ▸ provides a schematic summary of our method for polar Fourier *ab initio* 3D reconstruction.

### 2.4. Interpolation of polar common lines

Since each slice is discretized over 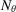 in-slice rotations on 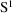, the line-extraction operation 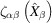 extracts the common radial line 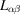 of slice 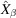. The common line lies at a continuous angular position 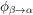 in slice 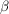. In general, this angle does not coincide with a discrete angular sample. Let 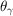 and 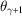 be the two adjacent angular coordinates in slice 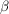 such that
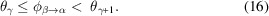
Rather than interpolating in angular coordinates, we perform interpolation in the embedded Cartesian Fourier plane. Let 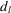 and 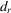 denote the Euclidean distances to the two neighboring angular samples. The interpolation weights are defined as
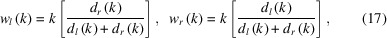
where the radial weighting proportional to the frequency index *k* corresponds to the Jacobian of the polar-to-Cartesian coordinate transformation, which ensures that contributions from higher-frequency rings are appropriately weighted. The interpolated radial line is given by
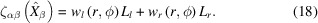
The extracted interpolated radial line is subsequently inserted into slice 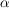 at its corresponding angular location using the same distance-based weighting scheme.

### 2.5. Point-group symmetry

For point-group symmetry 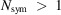, we incorporate symmetry directly in reprojection estimation by including symmetry-induced common-line contributions for every particle. For each particle orientation, all non-identity symmetry operators generate additional valid common lines with each reference slice; these radial lines are extracted and inserted in polar Fourier space during the averaging step. Symmetry is therefore enforced during the iterative reciprocal-space estimation, without constructing or symmetrizing a 3D volume.

### 2.6. Estimation of SSNR from polar reprojection correlations

The shell-wise prior variance 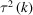 is estimated from an even/odd split of the dataset. Twofold cross-validation in our algorithmic context yields two sets of polar reprojection estimates, 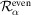 and 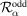, for each discretized projection direction 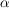. For each resolution shell *k* (corresponding to radial coordinate *r*), we compute a ring-wise Fourier correlation (Saxton & Baumeister, 1982 ▸) across all projection directions:
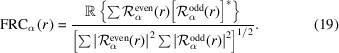
This quantity measures the reproducible signal content of the reprojection model at each spatial frequency. Under the assumption of independence between half-sets (Scheres & Chen, 2012 ▸; Sorzano *et al.*, 2022 ▸), the corresponding spectral signal-to-noise ratio (SSNR) is estimated using the standard relationship (Unser *et al.*, 2005 ▸)
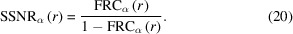
The ring-wise prior variance 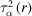 is then obtained directly from this SSNR estimate. Because the reprojection model is constructed entirely in the polar Fourier domain, this procedure provides a resolution-dependent estimate of signal variance without requiring the formation of intermediate 3D volumes.

### 2.7. Polar Fourier *ab initio* 3D reconstruction algorithm

We use the same optimization scheme as in our recently published *ab initio* 3D reconstruction algorithm based on probabilistic projection matching, described in detail by Van *et al.* (2025 ▸). The search is divided into eight stages (see Table 1 ▸). The degree of down sampling decreases with increasing stage number. The initial 3D orientations subjected to optimization are generated randomly. In the first two stages, regularization with a Gaussian low-pass filter is applied. In the following stages, maximum-likelihood (ML) regularization is applied to denoise the reprojection model. The regularization is limited to uniform Fourier filtering approaches (Gaussian filter and ML regularization). Non-uniform real-space regularization approaches are not applicable without additional transformation steps, since we operate exclusively within the polar Fourier domain. The optimization algorithm outlined in equations (14) and (15) is applied in each stage.

### 2.8. Ensemble *ab initio* 3D volume analysis

In the next section, we describe numerous repeated benchmarking runs of our method on a variety of single-particle datasets. To simplify the presentation of our results and to avoid having to show all the reconstructed density maps obtained from a given dataset, we implemented a tool for analysis of an ensemble of 3D volumes. The 3D orientation of an *ab initio* 3D map as well as its absolute hand (Rosenthal & Henderson, 2003 ▸) is arbitrary. Our method for 3D registration of arbitrarily oriented 3D density maps with unknown handedness relies on pairwise *ab initio* docking of all possible pairs of inputted volumes in their two respective hands, followed by calculation of a correlation-based metric of similarity, calculated in the resolution range [6, 100] Å, and stored in a matrix. Next, we analyze all pairwise correlation-based scores to determine which of the 3D volumes shows the best agreement to all the other ones. The identified medoid volume is the representative volume of the ensemble – the one with the largest sum of similarities to all other members. No averaging of volumes is done. Instead, the volumes of the ensemble are compared with the medoid volume, and possible outliers are identified through analysis of the correlation-based scores.

## 3. Results

### 3.1. *Ab initio* 3D map quality assessment

**Figure 3:**
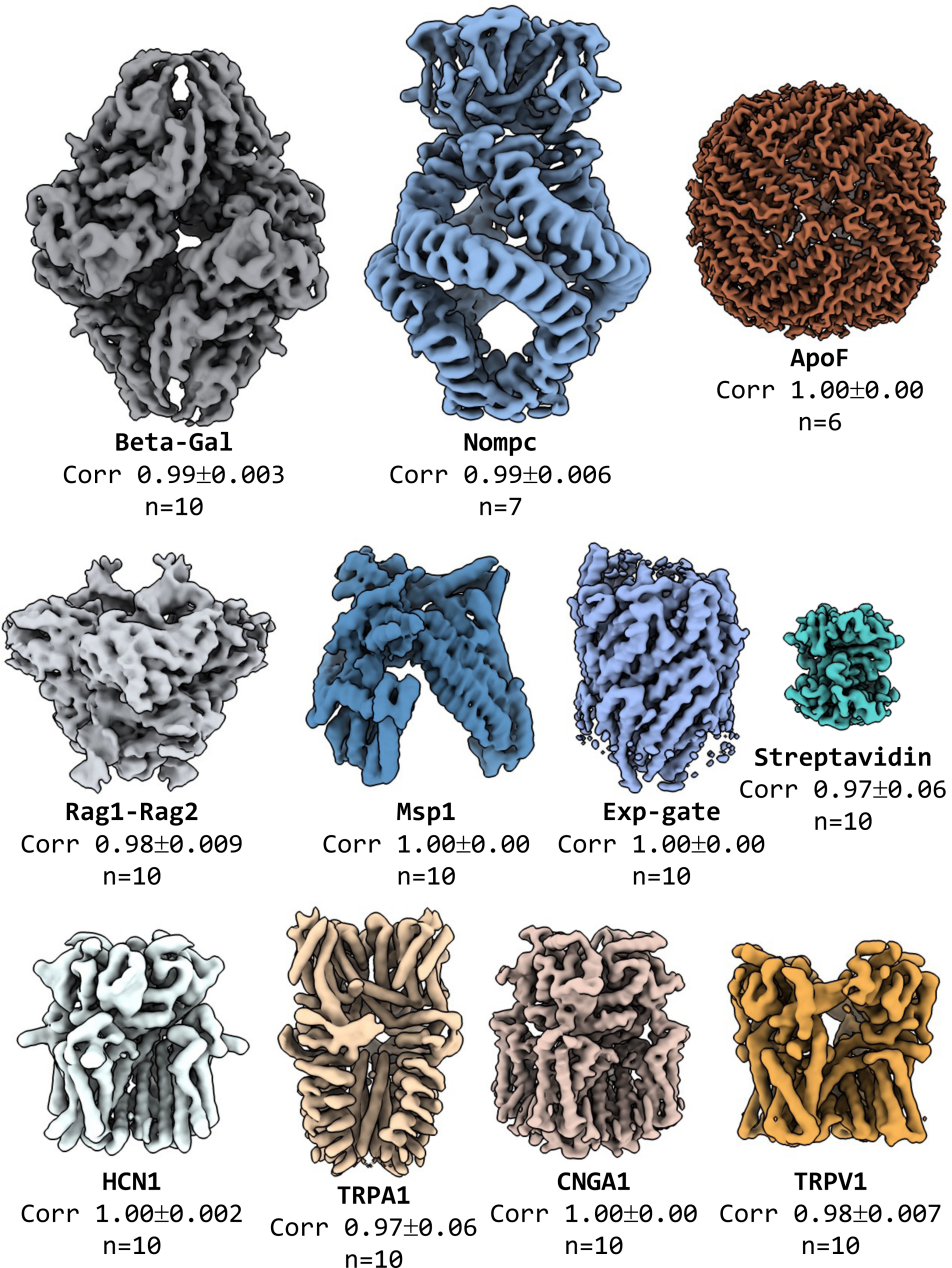
Three-dimensional maps obtained with the discrete polar Fourier *ab initio* 3D reconstruction approach. Number of independent repeats (*n*) are indicated together with the average correlation to the medoid of the ensemble, calculated to a resolution of 6 Å, and its standard deviation.

We sought to validate the ability of our algorithm to recover approximate relative particle 3D orientations and to reconstruct *ab initio* 3D volumes suitable for subsequent high-resolution refinement. Fig. 3 ▸ and Table S1 of the supporting information summarize the results for all analyzed datasets. Following particle import, 2D clustering was performed using the *SIMPLE* program *abinitio2D*, after which classes were manually selected. Table S1 reports, for each dataset, the number of retained particles used for 3D analysis, the success rate of the repeated *ab initio* 3D reconstruction runs and the resolution obtained.

The 2D analysis stage in *SIMPLE* (Caesar *et al.*, 2020 ▸) is a critical component of the workflow. Information extracted at this stage determines the degree of downsampling, the low-pass filter cutoff and the stochastic particle-update sampling used in the subsequent *ab initio* 3D reconstruction step, as previously described (Van *et al.*, 2025 ▸). During the *ab initio* 3D stage, particles were masked with a soft-edged spherical mask prior to computation of polar Fourier transforms. No *B*-factor sharpening or additional masking was applied to the reconstructed volumes derived from 3D orientations obtained using the polar Fourier *ab initio* method.

**Figure 4:**
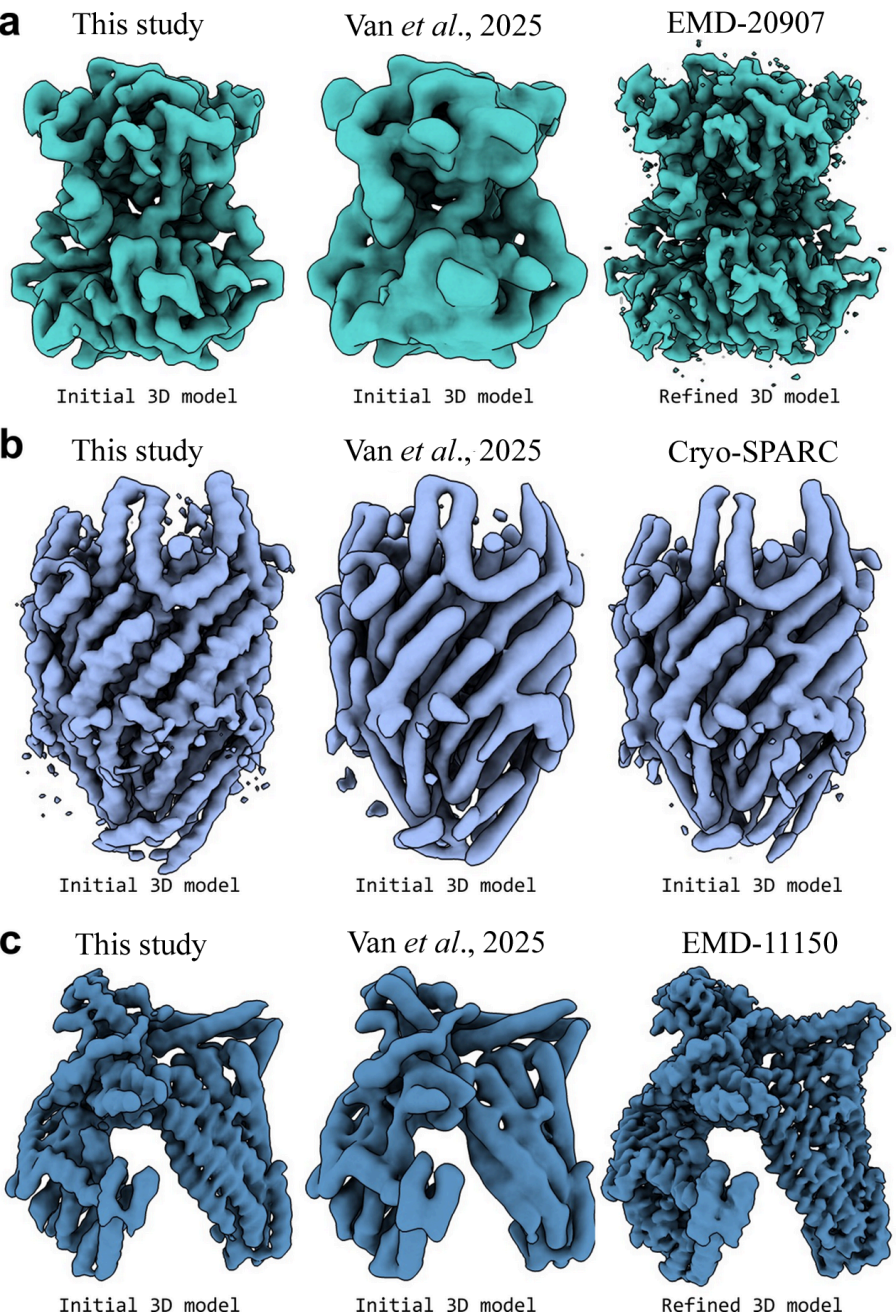
Comparison of *ab initio* 3D reconstructions. (*a*) 52 kDa streptavidin reconstructed using the method described in this study (left), volume-based probabilistic projection matching (middle) (Van *et al.*, 2025 ▸) and a map refined in *RELION* for comparison (right). (*b*) Asymmetric export gate reconstructed using the method described in this study (left), volume-based probabilistic projection matching (middle) (Van *et al.*, 2025 ▸) and an *ab initio* model generated with *cryoSPARC* for comparison (right). (*c*) Asymmetric main conformation of merozoite surface protein 1 reconstructed using the method described in this study (left), volume-based probabilistic projection matching (middle) (Van *et al.*, 2025 ▸) and a map refined in *cryoSPARC* for comparison (right).

Resolution estimates reported in Table S1 were computed using the FSC = 0.143 criterion and are expected to underestimate the resolution relative to volume-based Fourier shell correlation (FSC) estimates that employ envelope masking. Direct comparison with maps generated using volume-based probabilistic projection matching in our recent study (Van *et al.*, 2025 ▸) demonstrates equivalent or improved map quality for the polar Fourier *ab initio* approach. The most significant improvements are observed for lower molecular weight and lower symmetry targets, like streptavidin [Fig. 4 ▸(*a*)], export gate [Fig. 4 ▸(*b*)] and msp1 [Fig. 4 ▸(*c*)]. Critical methodological differences between the two approaches, in addition to the reprojection model, include the absence of envelope masking, the lack of real-space regularization and the removal of directional bias in the polar Fourier *ab initio* approach. The improved map quality is likely attributable to excessive real-space smoothing in the volume-based method (Van *et al.*, 2025 ▸), which was designed to generate an initial 3D model. In future work, we will re-design the non-uniform regularization method used in the volume-based approach and investigate opportunities for hybridizing the two methods.

### 3.2. Performance benchmarking

*SIMPLE* uses a stratified parallel execution model that is applied consistently across deployment environments. Fine-grained shared-memory parallelism (OpenMP) is implemented inside numerical kernels and domain operations, while coarse-grained multi-process parallelism is confined to application-level commanders that partition work, launch workers and aggregate results. Importantly, the same commander/worker model can be executed either (i) on high-performance computing systems using an abstracted job-queue interface (*e.g.SLURM*, *PBS*, *LSF*) or (ii) on a single node – such as a standard PC or Mac workstation or even a laptop – using a local backend that launches multiple worker processes directly. In all cases, the scientific workflow and control logic are identical; only the mechanism used to start worker processes differs.

The volume-based and volume-free 3D reconstruction applications considered here consist of three phases: probability-table generation and pose determination with partial reconstruction are done in distributed memory, whereas global aggregation/reduction of the distributed parts is done in shared memory (Van *et al.*, 2025 ▸). Both distributed-memory phases are embarrassingly parallel over the particle stack: the dataset is split into balanced subsets, each subset is processed independently by a worker process (using OpenMP threads within that process), and the outputs are then reduced into global structures used by subsequent steps. To quantify end-to-end performance, we measured the global wall-clock breakdown per iteration into these three phases, as well as the corresponding total time.

In the first stochastic search phase, each particle is processed independently, whereas in the second probabilistic phase, orientation assignments are coupled: assigning a particle to a given reference affects the probability landscape for all references (Van *et al.*, 2025 ▸). That coupling is what makes the method a truly global optimization scheme. Across all phases, most of the compute is spent on matching particles with the reprojection model. The second major bottleneck in the volume-based approach is the reconstruction and volume reprojection stages, which are considered jointly as ‘reprojection estimation’. The major performance advantage of the volume-free approach is in the reconstruction and volume reprojection stages: the 3D Fourier gridding and volume reprojection steps are replaced by a reprojection estimation directly on the polar grid. It is therefore of interest to compare directly the ‘reprojection estimation’ contribution with the total per-iteration wall-clock time, since the compute due to matching projections is identical between the two approaches.

**Figure 5:**
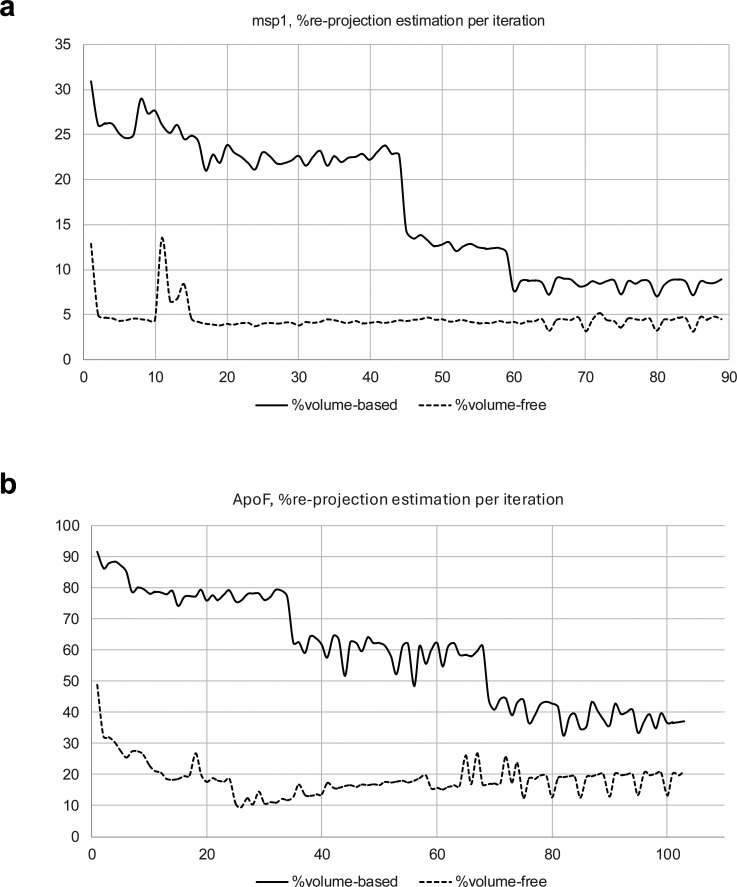
Fraction of per-iteration wall-clock time spent on reprojection estimation. Percentage of per-iteration wall-clock time attributed to reprojection estimation for the volume-based (solid) and volume-free (dashed) approaches. Reprojection estimation corresponds to 3D Fourier gridding and volume reprojection in the volume-based approach, and to direct estimation in the polar Fourier domain in the volume-free approach. (*a*) Msp1. (*b*) ApoF.

In the msp1 benchmark [Fig. 5 ▸(*a*)], reprojection estimation in the volume-based approach corresponds to 17% of the total per-iteration wall-clock time on average, whereas the volume-free approach only uses 5%. Overall execution times and number of iterations needed for convergence are presented in Table 2 ▸, indicating a best-case speedup of ∼1.8 and a worst-case speedup of ∼0.9 (a slow-down) for msp1. In the ApoF benchmark [Fig. 5 ▸(*b*)], reprojection estimation in the volume-based approach corresponds to 58% of the total per-iteration wall-clock time on average, whereas the volume-free approach only uses 19%. The 24 symmetry operations of the octahedral group increase the cost of reprojection estimation in ApoF relative to asymmetric datasets such as msp1. The overall speedup measured for the ApoF benchmark is 1.2 ± 0.06.

Overall performance gains correlate with the fraction of runtime spent on reprojection estimation in the volume-based approach; phases where projection matching dominates show limited speedup. Total execution times for representative datasets are summarized in Table 2 ▸.

All performance benchmarks were performed with a minimum of five repeats on a dual-socket Intel Xeon Gold 6242R workstation (2 × 20 cores, 2 hardware threads per core; 80 logical CPUs total; 3.10 GHz base frequency, 4.10 GHz Turbo; x86_64 architecture) with 252 GB RAM. The system was configured with two NUMA nodes. Hyper-threading was enabled and all 80 logical CPUs were utilized. The code was compiled using *GCC 15.2.0* with optimization flags -O3 -march = native -funroll-loops -fPIC. Ten distributed-memory partitions were used, each running eight OpenMP shared-memory threads in parallel. At most, 36 GB of RAM was utilized at any one time across all runs.

Although reprojection estimation with common lines is substantially faster than 3D Fourier gridding, acceleration of the full application is not guaranteed. For example, in the export gate and CNGA1 benchmarks the time spent on matching projections dominates and no speedup is observed. Our results demonstrate that while reprojection estimation can be significantly accelerated, the overall performance gain is limited by the projection matching, which dominates total runtime. Future optimization efforts will focus on accelerating the projection matching and exploring hybrid strategies that combine volume-based and volume-free updates to enhance convergence robustness.

## 4. Discussion

Efforts to detect common lines in large collections of noisy projection images typically lead to challenging optimization problems. Prior work approached this through combinatorial search, eigenvector methods and semidefinite programming, among other techniques (Singer & Shkolnisky, 2011 ▸; Singer *et al.*, 2010 ▸; Elmlund *et al.*, 2010 ▸; Elmlund & Elmlund, 2012 ▸; Muller *et al.*, 2024 ▸; Liu *et al.*, 2024 ▸; Herman & Kalinowski, 2008 ▸; Wang *et al.*, 2024 ▸). The approach we introduce here instead directly couples the manifold structure of the rotation group SO(3) (Agricola *et al.*, 2011 ▸) with the projection-slice theorem (Bracewell, 1956 ▸) through the polar Fourier transform (Baddour, 2019 ▸).

Rather than identifying common lines explicitly, we discretize SO(3) and represent each particle in a form that maps naturally onto this discretized rotational manifold, requiring no voting, ranking, dimensionality reduction, algebraic constraints or spherical embeddings. The resulting compact mathematical model provides a direct correspondence between particle orientations and the discretized rotation group, enabling the generation of reprojection estimates for arbitrary orientation configurations by averaging along lines in the discretized polar Fourier domain. A consequence of operating exclusively in reciprocal space is that real-space priors – such as solvent masking (Chen *et al.*, 2013 ▸), neural-network-based denoising (Kimanius *et al.*, 2024 ▸) or non-uniform regularization (Punjani *et al.*, 2020 ▸) – cannot be applied directly without additional transformation steps.

The ability of our method to produce high-quality *ab initio* 3D maps without any volume masking – beyond the spherical particle mask applied prior to polar Fourier transformation – is important for future integration into automated cryo-EM structure-determination pipelines. The resulting *ab initio* map quality distinguishes our approach from previous common-lines-based methods, which typically yield only low-resolution initial models. Moreover, the robustness of the optimization procedure is demonstrated by a single failed run on the 52 kDa streptavidin dataset out of 103 total runs across all datasets. This suggests that the optimization strategy deployed is well suited to the problem, although future work could explore whether slightly weaker but computationally faster optimization schemes might offer favorable trade-offs.

Both the present study and our recent work on probabilistic projection matching (Van *et al.*, 2025 ▸) build on the Bayesian framework for single-particle reconstruction established in *RELION* (Scheres, 2012*a* ▸,*b* ▸). That said, our implementations differ from a strict Bayesian derivation. We employ a renormalized likelihood objective to improve numerical conditioning, we replace weighted orientation marginalization with probabilistic hard-assignment optimization and we deploy importance sampling guided by the preceding 2D analysis step for accelerated 3D orientation search (Van *et al.*, 2025 ▸). These are deliberate design choices aimed at improving numerical stability, efficiency, convergence behavior and computational performance within the *SIMPLE* software environment.

Our overarching objective is to enable real-time cryo-EM image processing on commodity CPU hardware, providing statistical feedback about data quality during acquisition. This imposes practical constraints that can favor computational efficiency over strict Bayesian formalism. Our methods are therefore best understood as pragmatic adaptations that retain the core statistical principles of the Bayesian framework while restructuring the geometry and computational organization of the orientation-recovery problem to meet our engineering and performance objectives. The key distinction of our common-lines approach lies not in the statistical objective but in the computational representation of the projection and reconstruction operators.

There are several promising directions for further development of this methodology. One important avenue is to evaluate how the wide range of previously developed optimization strategies for 2D multi-reference alignment perform when augmented with common-lines constraints. Such investigations may give rise to a new class of methods capable of rapidly extracting 3D information at very early stages of data processing. The framework should also be extended to multi-volume *ab initio* 3D reconstruction, in analogy with the recent generalization of probabilistic projection matching (Van *et al.*, 2025 ▸). Finally, it remains to be determined whether jointly leveraging common-lines constraints and volume-based projection matching can improve the robustness of 3D orientation refinement for challenging datasets.

## 5. Conclusions

Our discrete polar Fourier formulation for single-particle 3D reconstruction is designed to meet the computational requirements of on-the-fly image processing during microscope data acquisition. In future work, we will implement and benchmark real-time versions of this algorithm to further enhance the live cryo-EM structure-determination capabilities of our *SIMPLE* software suite (Caesar *et al.*, 2020 ▸). While implemented within the *SIMPLE* framework, the formulation is general and applicable to any projection-matching or probabilistic orientation-recovery scheme.

*SIMPLE* is available for download at https://github.com/hael/SIMPLE.

## Supplementary Material

Table S1. DOI: 10.1107/S2052252526003611/rq5017sup1.pdf

## Figures and Tables

**Table 1:** Overall polar Fourier *ab initio* 3D reconstruction algorithm

Stage	Orientation search	Regularization	Shift search	Maximum No. of projection directions
1	Hybrid SHC/probabilistic	Gaussian	No	500
2	Hybrid SHC/probabilistic	Gaussian	No	500
3	Hybrid SHC/probabilistic	ML	Yes	1000
4	Hybrid SHC/probabilistic	ML	Yes	1000
5	Probabilistic	ML	Yes	1000
6	Probabilistic	ML	Yes	1000
7	Probabilistic	ML	Yes	1500
8	Probabilistic	ML	Yes	1500

**Table 2:** The total number of iterations (*N*_iters_) and total runtimes in hours and minutes for representative datasets *N*_sample_ is the number of particles subjected to update in each iteration according to our previously described importance sampling scheme (Van *et al.*, 2025 ▸).

Dataset	*N*_particles_	*N*_sample_	*V*_based_, *N*_iters_	*V*_free_, *N*_iters_	*V*_based_, total time (h, m)	*V*_free_, total time (h, m)
Streptavidin	8431	All	111 ± 6	107 ± 8	0h26m ± 2m	0h25m ± 4m
Msp1	112349	10000	89 ± 1	91 ± 3	3h31m ± 1m	2h58m ± 59m
Export gate	128994	5000	100 ± 2	113 ± 0	1h44m ± 1m	1h57m ± 1m
ApoF	200000	5000	113 ± 0	104 ± 0	4h15m ± 1m	3h33m ± 10m
CNGA1	225285	5000	94 ± 3	93 ± 1	3h6m ± 3m	2h51 ± 1m
