# Supplementary material for: A polar Fourier geometric approach to volume-free single-particle 3D reconstruction
Source: IUCrJ. 2026 Jun 11;13(Pt 4):354–63. doi: 10.1107/S2052252526003611 (PMC13324618; doi:10.1107/S2052252526003611)
Supplement: Supplementary file 1 [file m-13-00354-sup1.pdf]

IUCrJ

Volume 13 (2026)

Supporting information for article:

A polar Fourier geometric approach to volume-free single-particle 3D reconstruction

Cong T. S. Van, Cyril F. Reboul, Joseph J. E. Caesar, Rubén Meana-Pañeda and Hans Elmlund

Table S1

#	**Data set identifier**	**Point-group sym-metry**	**Molecular weight (kDa)**	**# particles inputted**	**# 2D classes**	**# particles selected**	**# *ab initio* repeats**	**Avg cc to medoid map**	**Succes rate (%)**	**Reso-lution @0.143 (Å) of medoid map**	**Map color**
1	EMPIAR-10335 (streptav)	D2	52	11,402	30	8,431	10	0.97±0.06	90	4.2	#4FB7B5
2	EMPIAR-10437 (msp1)	C1	225	141,838	300	112,349	10	1.00±0.00	100	7.7	#4F7FA8
3	Export gate (Kuhlen *et. al* 202)	C1	300	162,408	150	128,994	10	1.00±0.00	100	6.5	#8FA6D8
4	EMPIAR-10081 (HCN1)	C4	320	55,870	150	55,354	10	1.00±0.002	100	5.8	#DCECEC
5	EMPIAR-10722 (cnga1)	C4	320	274,333	200	225,285	10	1.00±0.00	100	5.2	#CDB3A8
6	EMPIAR-10005 (trpv1)	C4	400	35,645	150	25,016	10	0.98±0.007	100	6.6	#D39A4A
7	EMPIAR-10049 (rag1-rag2)	C2	400	108,544	250	95,510	10	0.98±0.009	100	5.4	#B8BCC2
8	Human Apoferritin (collected in-house)	O	480	1,083,636	200	200,000	6	1.00±0.00	100	4.0	#A96444

9	EMPIAR-10012 (bgal)	D2	500	5,513	90	4,716	10	0.99±0.003	100	5.9	#8E9094
10	EMPIAR-10024 (trpa1)	C4	500	43,585	250	31,948	10	0.99±0.004	100	7.2	#E1C2A2
11	EMPIAR-10093 (nompc)_	C4	780	175,314	300	169,753	7	0.99±0.006	100	7.4	#7FA0C8
